# Malaria elimination using the 1-3-7 approach: lessons from Sampov Loun, Cambodia

**DOI:** 10.1186/s12889-020-08634-4

**Published:** 2020-04-22

**Authors:** Soy Ty Kheang, Siv Sovannaroth, Lawrence M. Barat, Lek Dysoley, Bryan K. Kapella, Ly Po, Sokomar Nguon, John Gimnig, Rida Slot, Top Samphornarann, Seak Kong Meng, Gunawardena Dissanayake, Hala Jassim AlMossawi, Colleen Longacre, Neeraj Kak

**Affiliations:** 1 University Research Co. LLC, Phnom Penh, Cambodia; 2 National Malaria Control Program, Phnom Penh, Cambodia; 3 President’s Malaria Initiative/United States Agency for International Development, Washington, DC USA; 4 President’s Malaria Initiative/Centers for Disease Control and Prevention, Atlanta, Georgia USA; 5 Centers for Disease Control and Prevention, Atlanta, Georgia USA; 6 President’s Malaria Initiative/United States Agency for International Development, Phnom Penh, Cambodia; 7 University Research Co., LLC, Chevy Chase, MD USA

**Keywords:** Malaria, Malaria elimination, Surveillance, 1-3-7 approach

## Abstract

**Background:**

Cambodia has targeted malaria elimination within its territory by 2025 and is developing a model elimination package of strategies and interventions designed to achieve this goal.

**Methods:**

Cambodia adopted a simplified 1-3-7 surveillance model in the Sampov Loun operational health district in western Cambodia beginning in July 2015. The 1-3-7 approach targets reporting of confirmed cases within one day, investigation of specific cases within three days, and targeted control measures to prevent further transmission within seven days. In Sampov Loun, response measures included reactive case detection (testing of co-travelers, household contacts and family members, and surrounding households with suspected malaria cases), and provision of health education, and insecticide-treated nets. Day 28 follow up microscopy was conducted for all confirmed *P. falciparum* and *P. falciparum*-mixed-species malaria cases to assess treatment efficacy.

**Results:**

The number of confirmed malaria cases in the district fell from 519 in 2015 to 181 in 2017, and the annual parasite incidence (API) in the district fell from 3.21 per 1000 population to 1.06 per 1000 population. The last locally transmitted case of malaria in Sampov Loun was identified in March 2016. In response to the 408 index cases identified, 1377 contacts were screened, resulting in the identification of 14 positive cases. All positive cases occurred among index case co-travelers.

**Conclusion:**

The experience of the 1-3-7 approach in Sampov Loun indicates that the basic essential malaria elimination package can be feasibly implemented at the operational district level to achieve the goal of malaria elimination in Cambodia and has provided essential information that has led to the refinement of this package.

## Background

Malaria remains a leading cause of death and disease in many developing countries, with an estimated 219 million cases and 435,000 deaths occurring globally in 2017 [1]. The Southeast Asian Region has made significant progress in reducing its malaria incidence rate, experiencing a 59% decline in new cases from 2010 to 2017 [1]. The reduction in annual parasite incidence (API) is attributable to the malaria control program efforts with support from the Global Fund, USAID/PMI, as well as other non-programmatic factors including deforestation, climate change, improved infrastructure, etc. As a result, many countries in the region are moving toward malaria elimination. Cambodia has targeted malaria elimination within its territory by 2025 [2, 3], and has developed a malaria elimination package that includes strategies and interventions [4] designed to achieve this goal. Driving the push for malaria elimination is the intensification of artemisinin resistance and the development of multiple partner drug resistance in the western region of Cambodia.

**Fig. 1:**
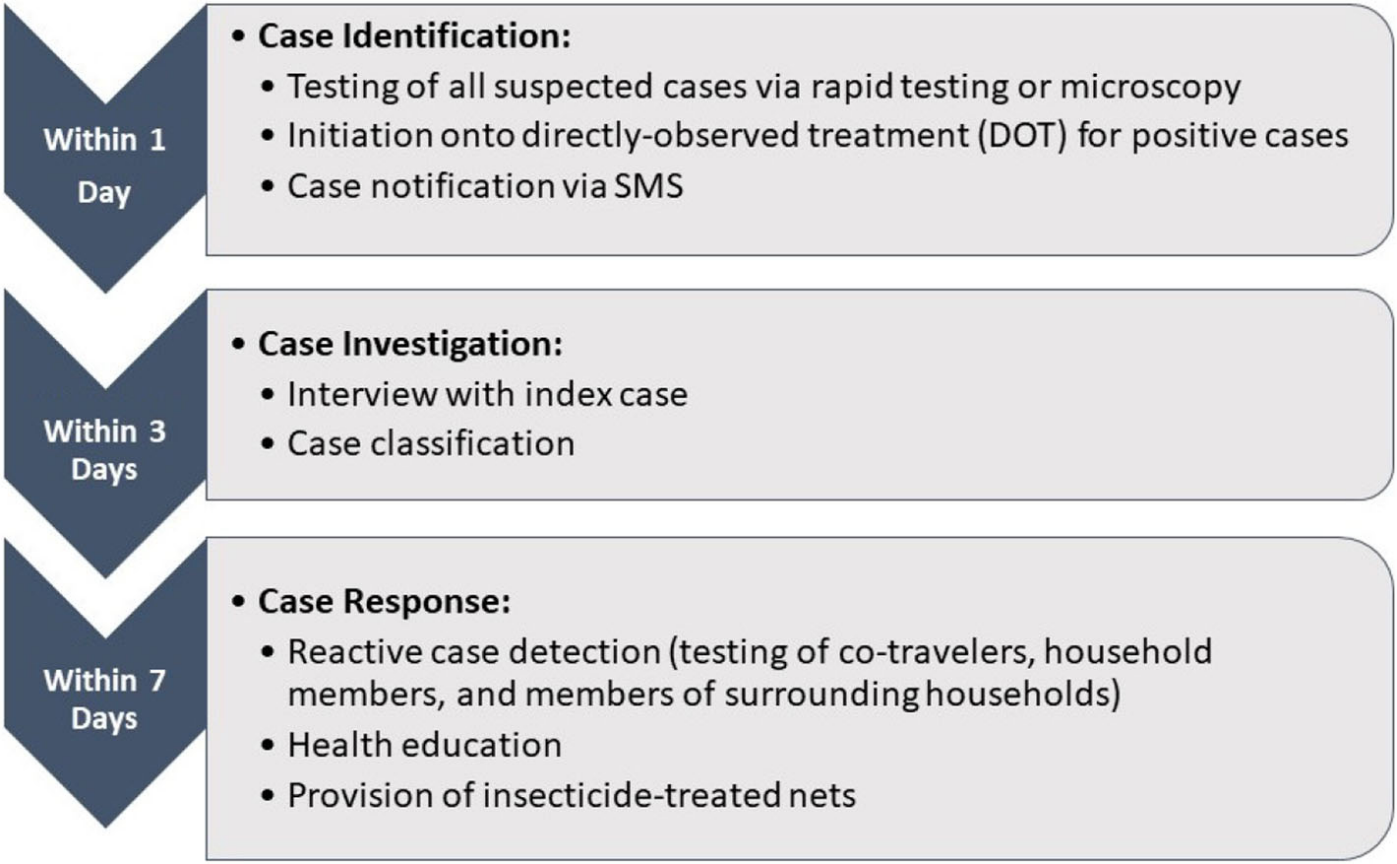
Overview of 1-3-7 Malaria Elimination Approach

A key part of the elimination strategy is the 1-3-7 surveillance and response model, which involves reporting of confirmed malaria cases within one day, investigation of malaria cases confirmed through rapid diagnostic testing (RDT) within three days, and application of targeted control measures to prevent further transmission within seven days (Fig. 1). The 1-3-7 strategy was initially developed and implemented in China in 2012 [5–7] and has since been adapted to the local contexts in several country settings in Southern Africa and Southeast Asia [8–10].

With support from the President’s Malaria Initiative (PMI), the United States Agency for International Development (USAID) Control and Prevention of Malaria (CAP-Malaria) Project supported national, provincial, and district health authorities in Cambodia to pilot and then scale-up a simplified 1-3-7 model in the Sampov Loun operational health district in Western Cambodia beginning in July 2015. The purpose of this article is to detail the experience in Sampov Loun in implementing the 1-3-7 elimination surveillance approach from July 2015 – January 2017 and to discuss challenges and lessons learned for potential future scale-up.

## Methods

### Selection of Sampov Loun

Sampov Loun is an operational district in Battambang Province in Western Cambodia with a population of approximately 160,000 people across three administrative districts and 127 villages. At the time of initial implementation, the health care infrastructure and capacity in Sampov Loun was comprised of nine health centers, one former district hospital, one referral hospital, 32 private providers, and 168 village malaria workers. Sampov Loun was targeted for malaria elimination as it experienced a significant decline in reported cases from 7.54 per 1000 population in 2012 to 2.87 per 1000 population in 2014, and it was identified as a site of intensifying artemisinin resistance. The overall objective of the program was to develop and implement an elimination model using the 1-3-7 approach within the existing public health system in Sampov Loun and to document the feasibility of the model.

### Components of the intervention

The cascade of care begins on Day 1 with suspected malaria cases being identified by village malaria workers or at health facilities. All suspected cases are tested via either RDT or microscopy. Patients with negative results are advised to seek consultation at public health facility. Patients with positive results are immediately placed on a three-day directly observed therapy (DOT) regimen. Uncomplicated malaria cases were treated with dihydroartemisinin-piperaquine (DHA-PIP) from July 2015 – January 2016. However, because some malaria cases did not respond to DHA-PIP, the regimen for uncomplicated malaria cases was switched to artesunate-mefloquine (ASMQ) beginning in early 2016. Pregnant women in their first trimester were treated with quinine. Those with treatment failure by the day 28 follow up were deemed to be drug-resistant and were treated with quinine plus tetracycline.

The health worker who made the diagnosis notified the malaria case to the district malaria coordinators using SMS from their mobile phones. Within three days of notification, case investigations were conducted by village malaria workers and health facility staff. Case investigations included interviews with the index case and resulted in case classification (plasmodium species and case origin). Interviewers collected information on the patient’s malaria history, recent travel and co-travelers of the index case, household members, and malaria prevention practices. A co-traveler was defined as a person who has been working, traveling, or staying outside of the home village with an index case in the past 3–4 weeks. Individual case investigation reports were collected and uploaded to a centralized malaria elimination database. Within seven days of notification, targeted response measures were undertaken (although these often happened within three days in conjunction with case investigation activities). Response measures included reactive case detection (i.e. testing of co-travelers, household contacts and family members, and surrounding households with suspected malaria cases), and provision of health education and long-lasting insecticide-treated nets (LLINs). Day 28 follow up microscopy was conducted for all confirmed *P. falciparum* and mixed-species malaria cases to confirm clearance of parasitemia.

### Management structure

The elimination program in Sampov Loun relied on a multi-sectoral collaboration between the Cambodia National Malaria Program (CNM), the Provincial Health Department, Operational District, Public Health Facilities, Private Providers and Village Malaria Workers. Provincial and District Special Working Groups for Malaria Elimination, consisting of health and non-health departments, uniformed services (i.e. army and police), private sector partners, and volunteers were formed to support the implementation of malaria elimination strategy. The program also relied on cross-border collaborations with neighboring Thailand to conduct patient investigation and follow up as well as to develop bilingual behavior change communication materials.

## Results

### Implementation of 1-3-7 approach

**Fig. 2:**
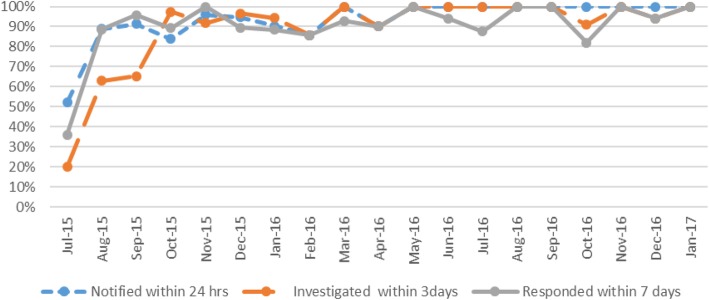
Summary of 1-3-7 elimination monthly surveillance and response results, July 2015 – January 2017

Figure 2 shows the percentage of malaria cases that were successfully notified within 24 hours, investigated within three days, and responded to within seven days. The percentage of cases notified within 24 hours rose from 50% in July 2015 to 100% in January 2017. Over the same time period, the percentage of cases investigated within three days rose from 20 to 100% and the percentage of cases responded to with targeted response measures rose from 35% to nearly 100%. Data from private providers was not collected from September – December 2017, due to changes in the national policy regarding the role of private sector providers in malaria control activities. Private providers are now instructed to refer all suspected malaria cases to public facilities for malaria diagnosis, treatment, and follow-up.

In response to the 408 index cases identified during the period of this pilot, 1377 contacts were screened (900 index household members, 395 co-travellers, and 82 surrounding household members), resulting in the identification of 14 positive cases (nine *P. falciparum* and five *P. vivax*). All positive cases were identified among index case co-travellers; there were no cases identified among index household members or surrounding household members. A total of 2492 individuals received health education and 242 LLINs were distributed. Rates of DOT provision gradually decreased from 86% (171/200) from July – December 2015 to 77% (99/128) from January – June 2016 and to 64% (51/80) from July 2016 – January 2017 because of an increase in loss to follow up due to high mobility and cross-border movement of those malaria patients.

### Malaria incidence

**Fig. 3:**
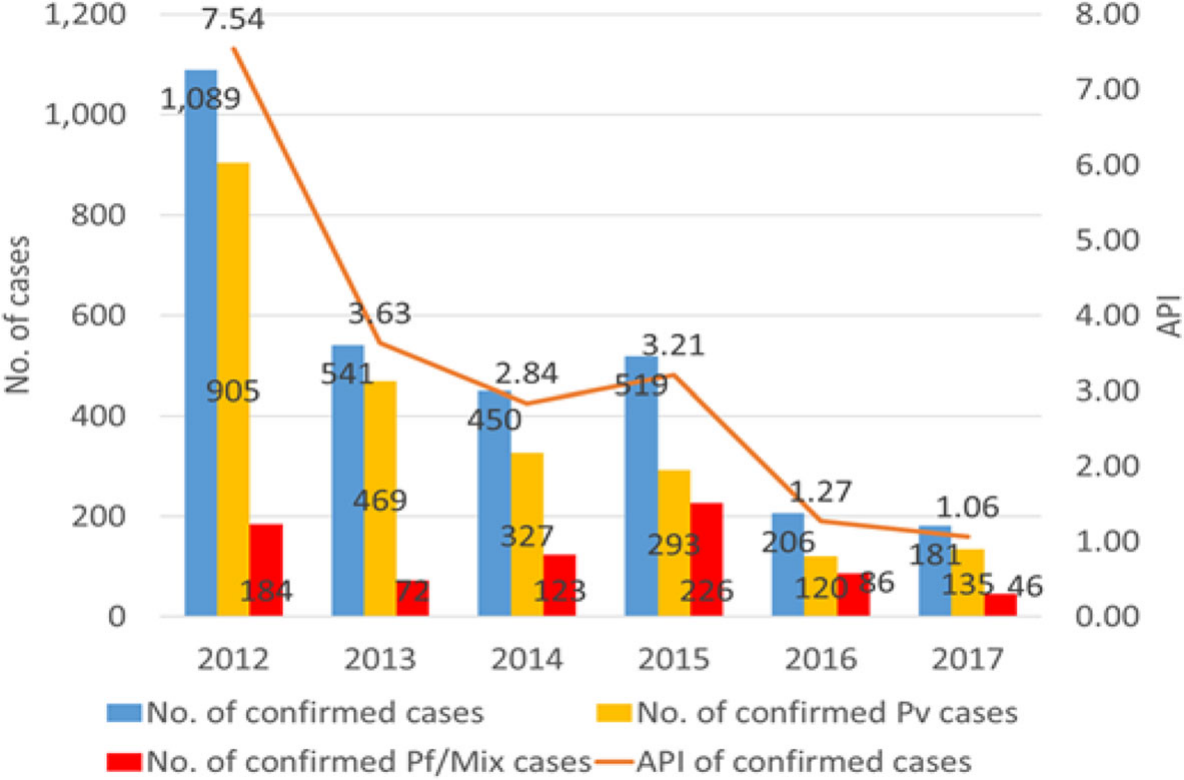
Yearly trend in confirmed malaria cases and annual parasite incidence (API) in Sampov Loun, Cambodia, 2012–2017

Figure 3 shows the overall trend in confirmed malaria cases in Sampov Loun from 2012 to 2017. Since implementation of the 1-3-7 elimination framework began in 2015, the annual parasite incidence (API) has fallen from 3.21 per 1000 population to 1.06 per 1000 population. Sampov Loun has also seen a steady decline in the number of confirmed *P. falciparum* and mixed *P. falciparum*/other species cases.

### Case classification

**Fig. 4:**
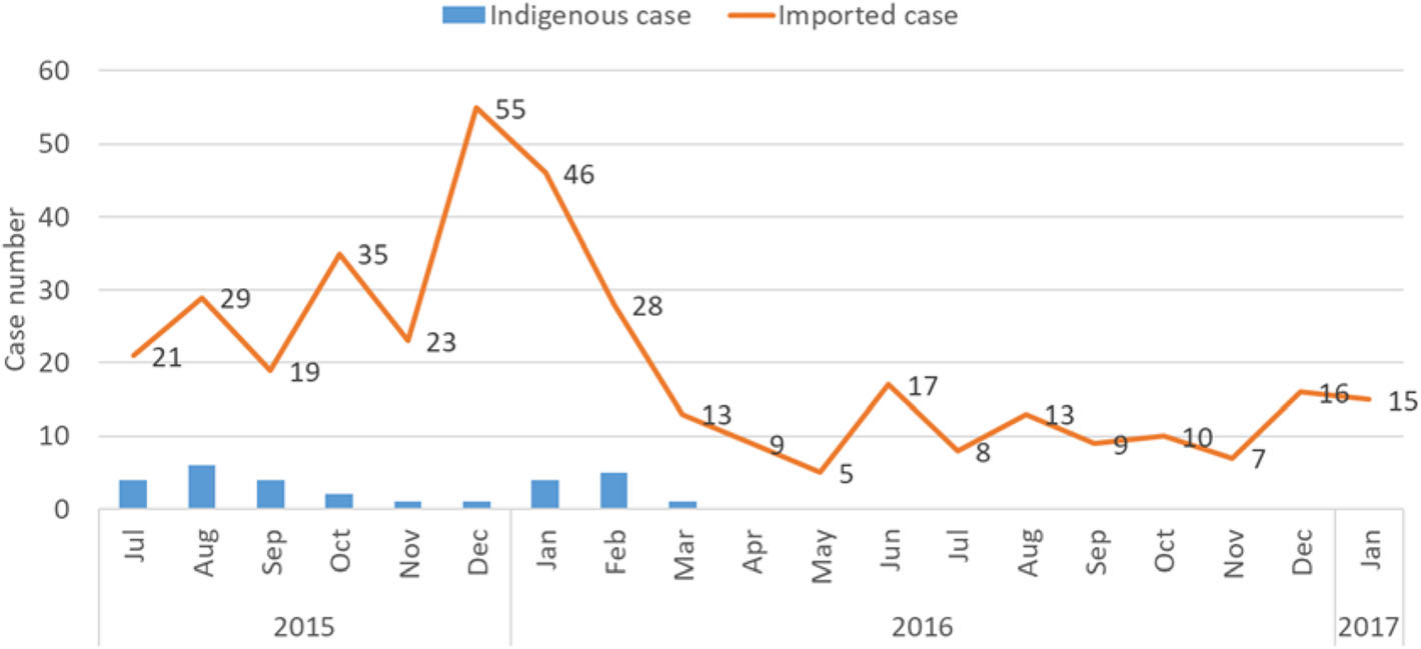
Mix of indigenous and imported malaria cases in Sampov Loun, July 2015 – January 2017

**Fig. 5:**
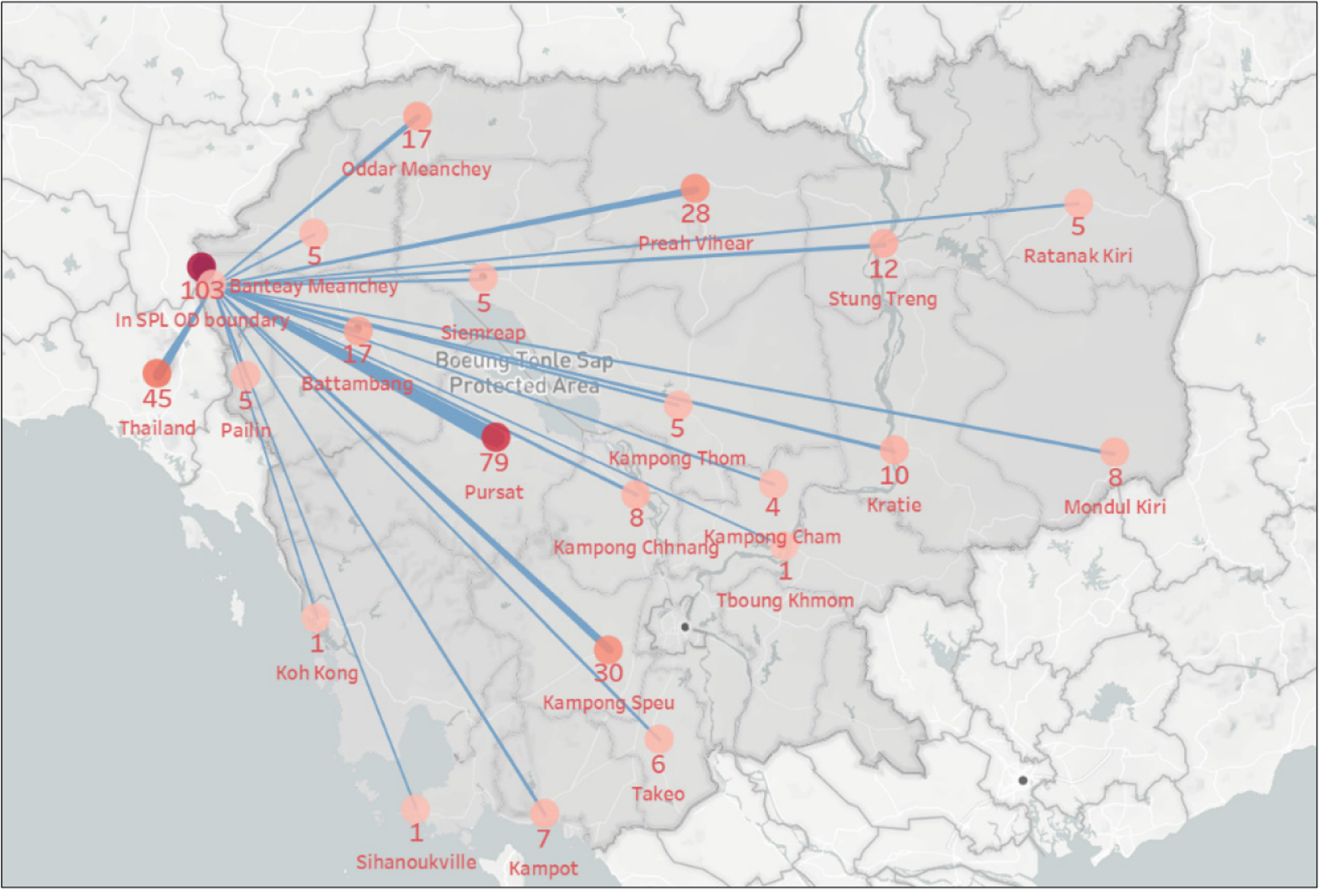
Origin of Imported Malaria Cases to Sampov Loun Operational District, July 2015 – January 2017

**Fig. 6:**
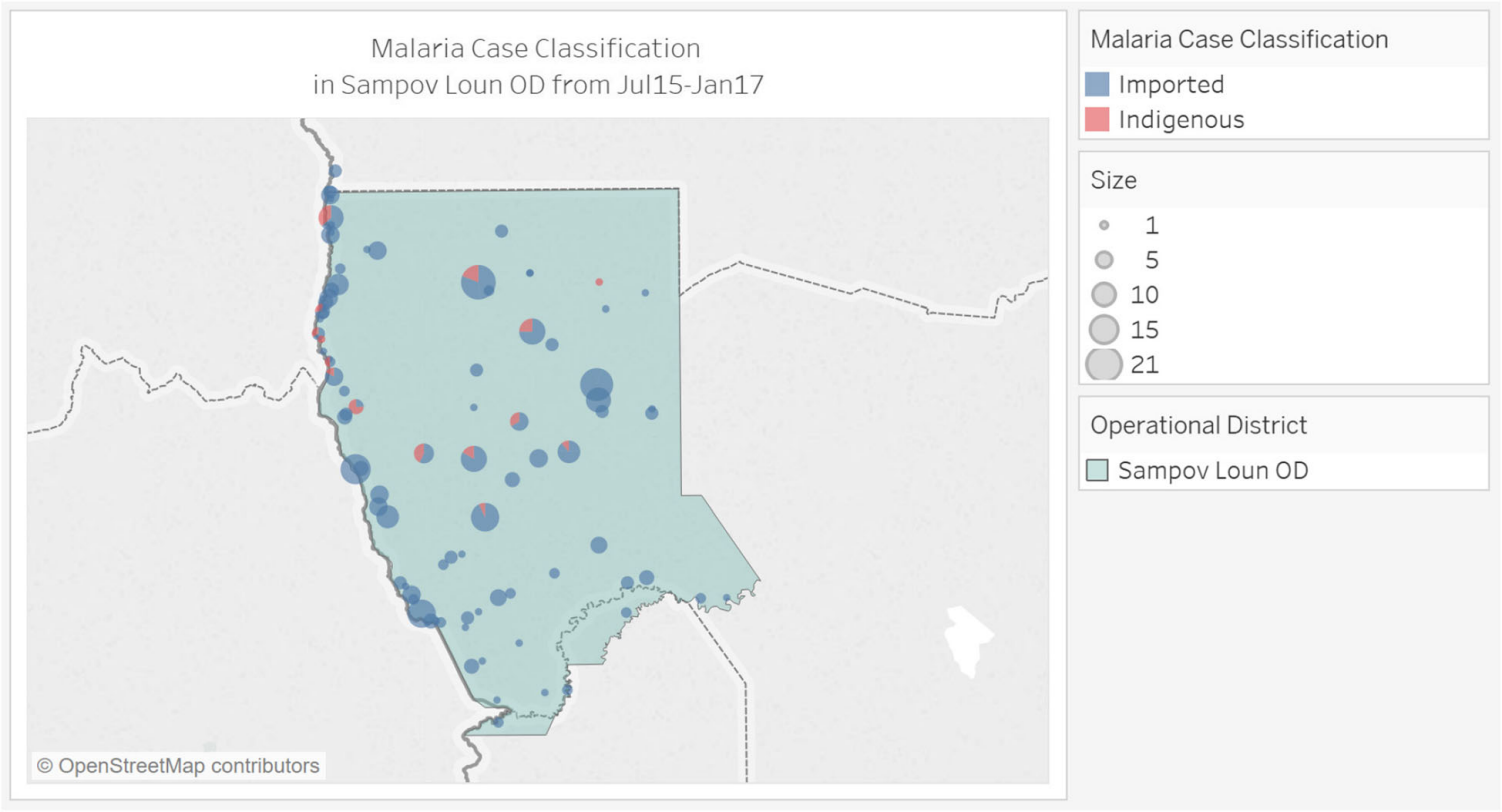
Malaria case classification, Sampov Loun, July 2015 – January 2017

Beginning in April 2016, all cases of malaria diagnosed in Sampov Loun have been classified as imported, indicating interruption of local transmission. While the district continues to see seasonal spikes in imported cases, these too are on a downward trajectory (Fig. 4). Case investigations have allowed Sampov Loun to track the origins of imported cases (Figs. 5 and 6). From July 2015 to January 2017, 11% of imported cases in the district were from Thailand, while 89% were from elsewhere within Cambodia. Of this 89, 31% were imported from neighboring provinces, while 69% were imported from other high-transmission areas, mostly in the eastern part of the country.

### Staffing requirements for malaria elimination

The malaria elimination activities in Sampov Loun were largely carried out by existing program staff, including OD Malaria Supervisors (ODMS) for supervision, surveillance, and case finding; laboratory technicians for reading of blood slides; and the Village Malaria Workers (VMWs) for early diagnosis using RDTs, treatment of malaria cases, providing DOT to confirmed malaria cases, and conducting reactive case detection among contacts of malaria index cases. The PMI provided LLINs, RDTs, and other consumables, which were distributed by the CAP-Malaria Project, as well as covered the costs of capacity building and supervision. The project also provided nominal transportation expenses to the VMWs/health facility staff to conduct household visits if needed. Every time a positive malaria case was notified, the health center staff and VMW visited the village within the first three days to undertake case investigation and plan for reactive case detection (RCD) within the first seven days. RCD efforts are being targeted on high risk groups such as co-travelers, forest goers and overnight stay in the forest fringe areas. The project is using SD Malaria Ag Pf/Pv RDT (Alere) to make diagnosis and to screen target populations. This RDT has sensitivity of 99.7% (98.5–100) and specificity of 99.5% (97.2–99.9%). In addition, the CNM team and Provincial Malaria Supervisor conducted supervision of health facilities and VMWs to ensure smooth implementation of the elimination activities. The RCD visits were used for screening households around the index case, co-travelers and household members to identify additional malaria positive cases. The overall costs of this model are minimal and thus the surveillance model is replicable with minimal additional support.

## Discussion

Results from the implementation of the 1-3-7 malaria elimination approach in Sampov Loun operational district from July 2015 to January 2017 demonstrate the feasibility of a local malaria elimination strategy, despite the challenges of multi-drug resistance and limited resources. The basic essential package of activities for malaria elimination, consisting of a combination of community-based case management and the 1-3-7 surveillance and response approach, was manageable at the community level by village malaria workers and local health facility staff with facilitation and support from local authorities in this very low incidence area. An entomology component, including indoor residual spraying and entomological survey, were not initially included in the package.

The integration of mobile health technology enabled Day 1 case notification via SMS messaging and helped improve real-time surveillance efforts. Practically, case investigation and response activities were often conducted simultaneously, resulting in what local officials called a “1-2-2 model” or “1-3-3 model” rather than a “1-3-7” model. Based on the experience of Sampov Loun, Cambodia has begun implementing this package in four neighboring operational districts (Battambang, Maung Russei, Thmar Koul, and Pailin) and is targeting other nearby provinces in Cambodia for initiation of these activities in the coming years. Reduced malaria burden in neighboring districts also catalyzed further success in Sampov Loun, as it reduced the risk of imported cases being reintroduced into the district.

The results from Sampov Loun generated valuable insights that can help make the 1-3-7 approach more efficient in the future. For example, in Sampov Loun, reactive case detection (RCD) efforts yielded very few positive results (0.9% positive rate over the period of implementation). Other studies have similarly questioned the efficacy of RCD in low transmission settings [11] or among neighborhood or hotspot contacts [12] and suggested that new approaches designed to optimize RCD are needed. In Cambodia, results suggest that focusing RCD efforts on co-travelers and forest worksites may be more effective than wider contact testing. Similarly, case investigations revealed that peri-domestic transmission was rarely, if ever, occurring in the district. This suggests that strategies that prevent peridomestic transmission, such as indoor residual spraying (IRS), may not result in additional malaria elimination gains. Finally, the 1-3-7 approach enabled the district to develop highly detailed maps of malaria cases, allowing for the identification of hot spots to be targeted for future activities.

Several challenges were identified during implementation. First, the program experienced declining motivation among health workers to pursue case investigation and contact testing, particularly during weekends and public holidays. Maintaining workforce motivation, as well as collaborations with private providers, is critical to ongoing elimination effort success. In addition, in a context where most cases are imported from outside the district, district-level response activities alone are likely to be ineffective in interrupting transmission. Communication and surveillance linkages with other operational district malaria response teams is necessary to sufficiently address external sources of infection [10, 13]. Reducing malaria burden in neighboring districts also has positive spillover effects, as the risk of re-introduction decreases. Strengthened cross-border collaborations are also needed to ensure adequate coverage of migrant and mobile populations with malaria preventive, diagnostic and treatment services [14, 15].

## Conclusion

The experience of the 1-3-7 approach in Sampov Loun indicates that the basic essential malaria elimination package can be feasibly implemented in operational districts with very low-level transmission to achieve the goal of malaria elimination. As a result of the successful implementation in Sampov Loun, Cambodia has scaled up elimination activities in all operational districts of Battambang Province and Pailin Province, and planning to expand activities to neighboring provinces, while continuing to target malaria elimination countrywide by 2025. The national malaria program is exploring the possibility of integrating 1-3-7 or a variant of this in its Malaria Elimination Action Framework for the 2020–2025 period.

## Data Availability

Data presented here is available at the health facilities that were supervising the malaria program in the project sites. Aggregated data are available upon request from the authors or from CNM.

## Abbreviations

API: Annual Parasite Incidence
AMQ: artesunate-mefloquine
CNM: Cambodia National Malaria Program
DHA-PIP: dihydroartemisinin-piperaquine
DOT: directly observed therapy
LLIN: long-lasting insecticide-treated nets (LLINs)
ODMS: Operational District Malaria Supervisors
PMI: United States Presidents Malaria Initiative
RCD: Reactive Case Detection
RDT: Rapid diagnostic tests
SMS: Short Messaging Service
USAID: United States Agency for International Development
VMW: Village Malaria Worker
